# Exploring the In Vitro Photoprotective Effect of a Combination of *Aspalathus linearis* Natural Extracts: First Steps in Developing New Technologies for Photoprotection Strategies

**DOI:** 10.3390/ijms26052330

**Published:** 2025-03-05

**Authors:** Irene Cáceres Estévez, Luisa Haya Rodriguez, Elena Haro Perdiguero, Francisco Javier Moreno Tovar, David Montalvo Lobo, Luis Nieto Botella, Salvador González, Ana López Sánchez

**Affiliations:** 1 Cantabria Labs, 28043 Madrid, Spain; irene.caceres@cantabrialabs.es (I.C.E.); luisa.haya@cantabrialabs.es (L.H.R.); elena.haro@cantabrialabs.es (E.H.P.); javier.moreno@cantabrialabs.es (F.J.M.T.); david.montalvo@cantabrialabs.es (D.M.L.); luis.nieto@cantabrialabs.es (L.N.B.); 2 Department of Medicine and Medical Specialties, Alcalá de Henares University, 28805 Madrid, Spain

**Keywords:** natural extracts, photoprotection, *Aspalathus linearis*, *Polypodium leucotomos*, fernblock, UV radiation, photobiology

## Abstract

There is a need for new technologies in photoprotection. The negative effects of solar exposure on the skin have been amply demonstrated and there is an urgency for the development of alternative photoprotective approaches. In this respect, natural extracts represent the most interesting and promising source. Among them, *Aspalathus linearis* extracts appear to be an excellent candidate due to supporting evidence, their multiple beneficial biological effects, and their robust toxicological profile. Here, we first explored the photoprotective properties of two different *Aspalathus linearis* extracts (fermented and unfermented) individually, and then in combination, in a simplified model assessing Normal Human Dermal Fibroblast survival after UVB radiation. Surprisingly, we found the fermented extract to be more photoprotective than the unfermented one. In addition, a specific combination of the two extracts showed a synergistic effect. By HPLC and FRAP analyses, we observed that the photoprotective effect did not correlate with the amount of *Aspalathus linearis* main characteristic metabolites nor with the antioxidant capacity of the ingredients. Finally, an additional photoprotective effect was observed when *Aspalathus linearis* extracts were combined with a *Polypodium leucotomos* extract (Fernblock^®^), a well-known botanical ingredient with demonstrated photoprotection activities. Thus, this work provides a solid scientific basis for the inclusion of this technology in future topical and systemic photoprotective strategies.

## 1. Introduction

Sun radiation is one of the most dangerous environmental stressors conditioning life on the Earth’s surface [1,2]. In fact, excessive exposure to solar radiation has been widely demonstrated to have important detrimental effects on skin health, including DNA damage, carcinogenesis, inflammation, immunosuppression, and photoaging [3]. The solar spectrum can be divided into different wavelength ranges, with ultraviolet radiation B (UVB) being considered the most direct hazard for skin tissues [4], although there is increasing evidence that other wavelengths can significantly affect skin health as well [5,6]. Over the past few decades, advances in photobiology have enabled the development of effective photoprotection strategies [7] which can help prevent, among others, skin cancer [8]. In this context, increasing knowledge about the sun’s effects on the skin, the development of innovative prophylactic technologies, and of course, society’s beauty trends, health awareness, and toxicity concerns, have all progressively shaped the evolution of approaches to photoprotection [7,9].

Currently, experts in the field indicate that the future of photoprotection is increasingly moving towards personalized protocols and regimens that take into account the vast array of intrinsic and extrinsic elements determining the specific necessities of each individual, and then subsequently identifying the most suitable photoprotective tools accordingly [7,10,11]. To enable the implementation of these strategies, the different technologies and their associated modes of action should be considered. At present, the main photoprotective measures are related to the avoidance of solar irradiation by seeking shade, use of photoprotective clothing, hats and glasses, and sunscreens [12,13]. As part of these measures, there are many different sunscreens where solar filters are of primary importance. These filters reflect or absorb detrimental radiation, preventing it from reaching cells and causing damage [14]. That is the reason why sun filters are currently the main photoprotective ingredient in sunscreens.

However, in addition to the traditional use of topical measures, systemic ingredients are progressively making their way into photoprotection strategies. While relatively new and still needing further evidence to support their use in monotherapy, there is robust evidence supporting their important role in complementing photoprotective strategies in combination with topical measures [15], especially in vulnerable populations [16].

In this case, the mode of action of most ingredients used in systemic approaches (and some ingredients used in both topical and systemic strategies) involves the modulation of the cell’s endogenous protective mechanisms. As life on the Earth’s surface has developed in a high-radiation environment, human cells naturally feature different mechanisms for protecting and repairing radiation-induced damage. Thus, in addition to modifying the amount of radiation reaching the cells, some technologies are able to enhance these cells’ natural defenses [17].

The different ingredients and modes of action can be more or less appropriate depending on skin type and condition, exposure conditions, age, lifestyle, etc. To ensure optimal protection based on individual needs, this should be taken into consideration when designing personalized photoprotection strategies [7]. The more tools and technologies that can be considered for combination and integration into photoprotective regimens, the better, justifying the pressing search for new ingredients.

In this scenario, natural extracts are one of the most interesting and promising sources of photoprotective agents [18,19]. On one hand, some organisms produce molecules, such as specific flavonoids and mycosporine-like amino acids, capable of absorbing ultraviolet radiation (UVR), the most harmful part of the solar spectrum, and are, thus, of great interest for the development of natural filters [20,21,22]. On the other hand, extracts of natural origin can also be rich in different compounds with antioxidant properties. This is especially relevant as the ROS (Reactive Oxygen Species) induced by solar radiation are one of the key events in mediating subsequent deleterious effects on skin cells and tissue [23], and antioxidant compounds have the potential to buffer ROS production [24]. In addition, although sun filters can provide excellent broad spectrum and stable protection, some can contribute to increasing the pool of radiation-induced ROS [25,26]. Thus, it has been proposed that combining filters with antioxidant compounds could maximize filtering capabilities while minimizing the production of ROS [27]. Supporting the benefits of including antioxidants of natural origin in complex sunscreen formulations are also reports of increases in the filtering capabilities and photostability [22,28]. Moreover, some natural extracts rich in antioxidants are able to stimulate the cell’s endogenous mechanisms against solar-induced damage, proving to be useful in topical and systemic photoprotection approaches [17,29]. All these potential benefits, along with a good acceptance from the general public and the belief that they can be less harmful to the environment, have encouraged a very active search for ingredients of natural origin with photoprotective potential [30]. Among these natural sources, the South African bush *Aspalathus linearis* has recently demonstrated very promising properties [31,32].

*Aspalathus linearis* (Brum.f.) Dahlg. is an endemic bush from the Cederberg region in South Africa [33], with important antioxidant, anti-inflammatory, antimutagenic, anticarcinogenic, antidiabetic, cardioprotective, and neuroprotective properties [34]. Those biological activities are due to its composition, rich in polyphenols and, in particular, specific flavonoids. Of these mentioned flavonoids, C and O-glycosides with remarkable antioxidant activities are key. Of particular interest are three dihydrochalcones: aspalathin and linearthin [32], specific to the species (*A. linearis*); and nothofagin (found in *A. linearis* and species from the *Nothofagus* genus) [35]. *A. linearis* extracts also contain less specific isomeric flavones such as orientin, isorientin, and isovitexin [36], lower proportions of other flavonoids such as luteolin, and the flavonols quercetin, isoquercitrin, and rutin [36]. Importantly, the compounds of interest are not usually standardized in the different forms of *A. linearis* extracts. For its traditional use as a tea, leaves and shoots from the bush are subjected to cutting, bruising, wetting, and sun drying, in heaps in the open air (38–42 °C), a process in which the plant tissues become dry and oxidized (known as “fermentation”) [37]. During this process, many of the previously cited components that form part of the fresh plant tissue are transformed [38,39] by enzymatic and/or chemical reactions [39,40,41]. While allowing for variability due to differences in farming and fermentation processes, it is generally accepted that the amount of aspalathin and nothofagin is high in fresh tissue, but significantly decreases during fermentation, being little present in fermented *A. linearis* extracts [36]. Two main types of *A. linearis* extracts can be identified: the traditional type subjected to the fermentation process, known as “fermented” (hereinafter ALF, from *Aspalathus linearis* fermented); and the “unfermented” extract (ALU, from *Aspalathus linearis* unfermented), obtained directly by drying the bush tissues. Due to the strong antioxidant properties of aspalathin and nothofagin, it is generally accepted that ALU extracts possess a much higher antioxidant capacity than ALF. For example, ALU showed 28% total radical trapping antioxidant parameter (TRAP) and 26.8% ferric reducing antioxidant power (FRAP), higher than ALF, in in vitro measurements [42,43]. Accordingly, most in vitro studies ascribing important biological activities to *A. linearis* have used ALU extracts, detecting or assuming much higher bioactivity in ALU than ALF, and correlating the biological benefits with the antioxidant levels [44,45]. This is the case of the photoprotective effect shown for *A. linearis*, where ALU extracts have been reported to act as cytoprotectants when applied prior to UVB irradiation in in vitro cellular models; the presence of the characteristic dihydrochalcones previously mentioned [32] and the antioxidant properties of the extracts are associated with both. This interesting photoprotective effect seems to be accompanied by anti-inflammatory [31,46] and proapoptotic cellular responses, which result in chemopreventive [47] and tumor promotion inhibitory properties [48]. Even though ALF generally features lower concentrations of the compounds of interest, and much lower antioxidant activity as compared to ALU, its water extractions (similar to traditional tea and with no reported photoprotective effects) have a strong safety profile supported by a widespread consumption, which could explain why, up until now, most clinical studies exploring the benefits of *A. linearis* have focused on this extract [49,50,51]. No research addressing the photoprotective properties of ALF has been performed to date; and even though certain cosmeceutical potential of *A. linearis* extracts have been suggested [52,53,54], we are not aware of any actual photoprotective technology based on these natural extracts [30].

Thus, this work aimed to explore the photoprotective effect of both *A. linearis* extracts. Accordingly, we first established a high-throughput experimental model using Normal Human Dermal Fibroblast (NHDF) cultures subjected to UVB radiation, which allowed us to test the photoprotective effect of the different extracts. Applying this cellular model, we observed a clear photoprotective effect (evidenced by increases in relative cell viability) when the cultures were pre-treated with aqueous extracts of ALU, mimicking previously published results in different experimental models [32]. Interestingly, ALF extracts also conferred higher levels of photoprotection, which could not be ascribed to the presence of *A. linearis’* most characteristic metabolites. In light of those results, we identified a specific combination of both extracts, which provided a significant synergistic photoprotective effect. The enhanced photoprotection observed was not associated with the antioxidant properties of the extracts. Finally, in an attempt to provide even greater photoprotection, we studied its performance when combined with Fernblock^®^, another botanical extract, derived from the fern *Polypodium leucotomos* (PLE) with extensively studied photoprotective properties [55]. In this assay, we observed a complementary effect, supporting the potential of the technology to be incorporated in current photoprotective strategies.

This study contributes to the characterization of the photoprotective activity of two different *Aspalathus linearis* extracts and their combination, and lays the scientific foundations for a new derived photoprotective technology. The encouraging results obtained in a specific cellular model open the door to further translational work, aiming to position this technology of natural origin as a new player to be considered in future alternative photoprotection strategies, making them increasingly personalized, effective, and safer.

## 2. Results

### 2.1. Photoprotective Properties of Two Different Aspalathus linearis Extracts (ALF and ALU)

To explore the photoprotective properties of ALF and ALU extracts, an experimental model based on the analysis of cell survival after UVB irradiation was developed using NHDF cell cultures (Figure S1). Considering previous results reporting anti-proliferative and pro-apoptotic properties in different cellular models [47], the effect of ALF and ALU extracts per se in NHDF was first addressed, confirming the absence of a cytotoxic effect in this model (Figure S2). To set up the experimental model, the survival rates of non-treated fibroblasts were analyzed under different doses of UVB (irradiation test), finding a significant reduction in cell survival under increasing doses of UVB *(*Figure S3). Each time a new culture was thawed, irradiation tests were performed, selecting radiation doses reducing cell survival from 40 to 60% for the following analysis. Then, to calculate the contribution of each treatment to induced photoprotection, the survival of the control non-treated UV-irradiated samples was considered “no induced photoprotection”. Thus, the increase in survival rates of treated groups and the control non-treated group was expressed as “Relative Photoprotection Over Control”.

**Figure 1:**
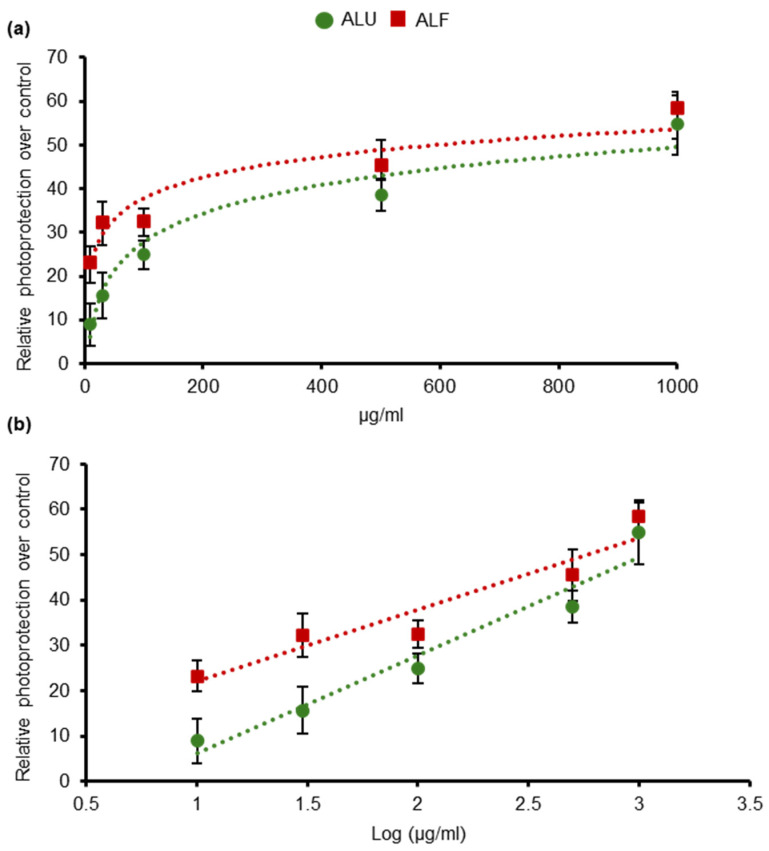
Photoprotective effect of ALU and ALF aqueous extracts. The X-axis represents the concentration of the extracts used in the treatments, expressed in linear scale in graph (**a**); and in a logarithmic scale in (**b**). The Y-axis represents the average values of relative photoprotection measurements. Survival of non-treated samples was 54.75%. UVB irradiation dose = 0.8 J/cm^2^. The different extracts were tested at 10, 30, 100, 500, and 1000 μg/mL. For all data points, *n* ≥ 4 independent wells of cells. Error bars represent the standard error of the mean (*SEM*). Data trendlines are drawn.

Consistent with the evidence from previous reports [32], ALU showed a clear dose-dependent photoprotective effect (Figure 1 and Figure S4a,b, and statistical analysis in Table S1, two-way ANOVA). Surprisingly, ALF also displayed a strong protective effect, presenting a higher photoprotective capability than ALU (Figure 1 and Figure S4c,d, and statistical analysis in Table S1, two-way ANOVA, *p*-value = 0.006).

Thus, under the conditions established in our cellular model, the results unveil, for the first time, the photoprotective character of ALF.

### 2.2. Analysis of ALF and ALU Characteristic Phenolic Compounds

*A. linearis* biological functions have been commonly ascribed to the presence of certain metabolites and their associated antioxidant capacity [32,34,56]. However, some of those compounds seem to be processed during the fermentation of the plant tissue, being less abundant in ALF extracts [36,39], and correlating with a lower antioxidant capacity [42,43]. This led to the consideration that for many of the previously reported biological benefits, ALF extracts were less effective than those from ALU [44]. Considering our previous results, in which we observed higher photoprotective activity for ALF extracts as compared to ALU, we analyzed the composition of the extracts used in this study for some of the flavonoids reported as characteristic compounds.

It is worth noting that as shown in Table 1, ALF featured 62.5 times less aspalathin, 21.25 times less nothofagin, 3.96 times less orientin, 1.86 times less isoorientin, 4.75 times less vitexin and 1.7 times less isovitexin when compared to ALF. Details of the calibration curves obtained for the quantification of phenolic compounds in ALU and ALF extracts are shown in Table S2.

As higher concentrations were observed in ALU vs. ALF extracts for most of the metabolites but higher photoprotective activity is conferred by ALF, a direct association between the photoprotective effect observed for ALF and the specific metabolites previously proposed cannot be established.

### 2.3. Enhanced Photoprotective Effect of a Specific Combination of ALU and ALF Extracts

Keeping in mind that, in our experimental model, both extracts conferred photoprotection, that other biological properties have been ascribed to these extracts [34], and that the combination of different natural extracts can occasionally produce additional effects [57,58], we decided to explore the photoprotection capabilities of ALU and ALF in combination.

By using the previously described experimental model, different extract combinations were tested for their photoprotective capabilities. For the sake of comparison, in all cases, the same final concentration was assayed for ALF and ALU independently, reproducing the results presented in Figure 1 (Figure S5).

**Figure 2:**
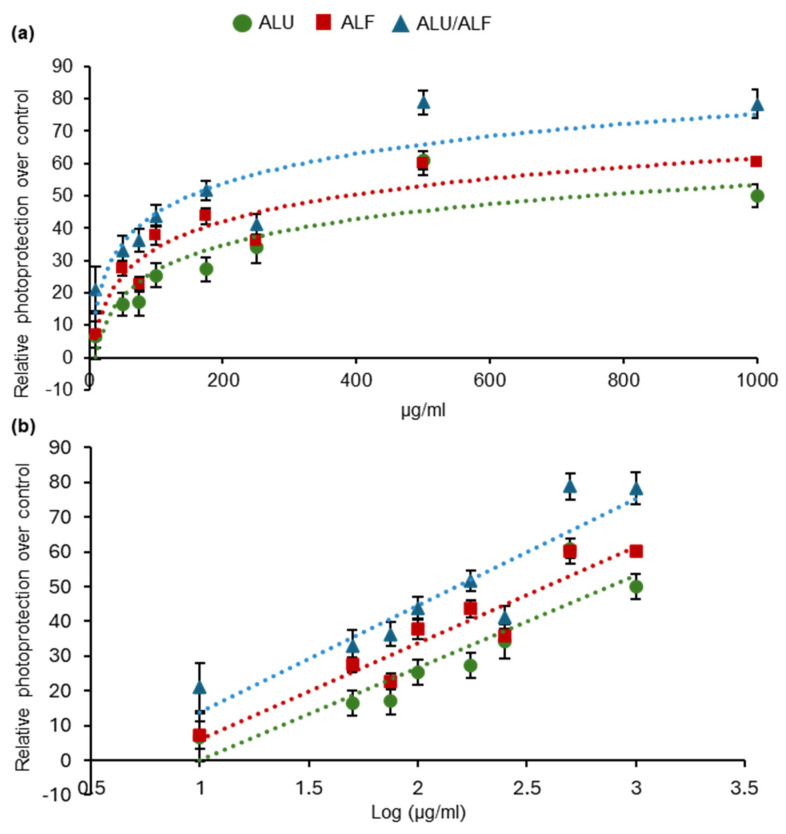
A specific ALU/ALF combination shows an enhanced photoprotective effect. The X-axis represents the concentration of the extracts used in the treatment for ALU (green circles)*,* ALF (red squares), and a specific combination ALU/ALF (blue triangles). Concentrations are in a linear scale in graph (**a**) and a logarithmic scale in (**b**). Each data point represents average values for the photoprotective effect shown in the fibroblasts (Y-axis) when pretreated with the specified concentration (X-axis). Survival of non-treated samples was 41.51%. UVB irradiation dose = 0.8 J/cm^2^. The different extracts were tested at 10, 50, 75, 100, 175, 250, 500, and 1000 μg/mL. For all data points, *n* ≥ 4 independent wells of cells. Error bars represent the standard error of the mean (*SEM*). Data trendlines were drawn.

As shown in Figure 2 and Figure S5, we identified a specific combination of ALU/ALF extracts (patent pending) providing a greater photoprotection capability than either ALU or ALF on their own (statistical analysis in Table S3, two-way ANOVA multiple comparisons, post hoc Tukey).

### 2.4. Comparison of ALU, ALF and ALU/ALF Antioxidant Properties

As mentioned before, some *A. linearis* properties have been ascribed to the plant’s composition and its associated antioxidant properties [34,59]. Thus, in order to identify the causes of the observed photoprotective properties, and after discarding a correlation with some of its most characteristic metabolites, we evaluated the antioxidant capacity using FRAP analysis.

**Figure 3:**
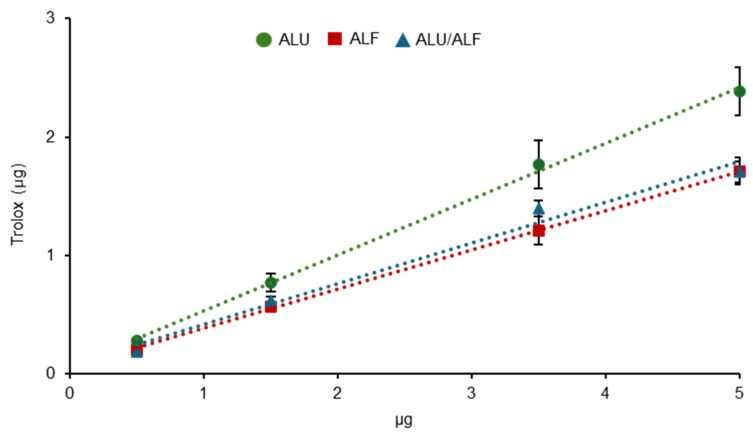
Antioxidant capacity of ALU, ALF, and ALU/ALF analyzed by ferric ion reducing antioxidant power (FRAP). Trolox is used as a reference antioxidant (positive control). FRAP absorbance measures were interpolated in Trolox linear equation to express the antioxidant capacity of the extracts relative to Trolox (μg of Trolox). The X-axis represents the amounts (μg) of the extracts tested. For all samples, *n* = 3 independent wells of cells. Error bars represent the standard error of the mean (*SEM*).

The FRAP measures obtained were higher for ALU than for ALF and the specific combination of ALU/ALF (Figure 3 and Figure S6). In this assay, Trolox was used as an antioxidant-positive control (Figure S6a). Specifically, ALU showed an average antioxidant capacity of 51.31% relative to Trolox (using the concentrations measured in this assay), while ALF showed one of 36.95%, and the combination ALU/ALF showed one of 38.3% (Table S4). Thus, ALF and the proposed combination ALU/ALF present approximately 27.99% and 25.36% lower antioxidant capacities than ALU as measured by FRAP (Table S4).

The results for ALF and ALU reproduced the data previously obtained by other groups [43,59]; however, this antioxidant capacity does not correlate with the higher photoprotective effect shown by ALF and ALU/ALF.

### 2.5. Additional Photoprotective Effect of ALF/ALU and Fernblock^®^

Photoprotection products often contain a combination of different active ingredients as beneficial effects have been reported for various combinations [14,60]. In order to characterize the performance of ALU/ALF in combination with other photoprotectants, and taking advantage of our experimental setup, we analyzed the activity of its combination with an extract of *Polypodium leucotomos* (PLE, commercialized as Fernblock^®^), another botanical ingredient widely used in both oral and topical photoprotective approaches [55].

**Figure 4:**
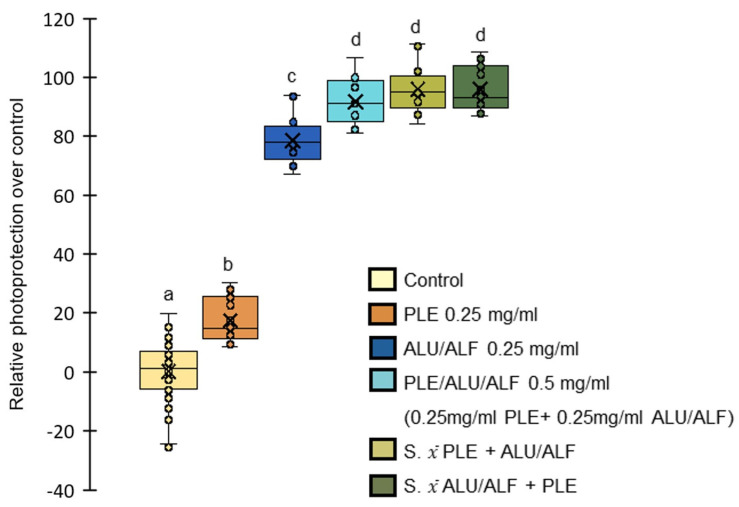
ALU/ALF and PLE incremental effect in photoprotection. The box and whisker plots represent the photoprotective effect shown in fibroblasts. Boxes represent the interquartile range (50% of the data around the median, Q1–Q3), median is marked as a line inside the boxes (Q2). Mean is expressed as “X” inside the boxes. Whiskers extend to each dataset’s maximum and minimum value within 1.5 times the interquartile range. Middle data points are overlapped on the boxes as opened circles. The yellow box represents the data for non-treated control samples. The orange box represents the data for PLE pretreatments at 0.25 mg/mL. The dark blue box represents the data for pretreatments with the specific combination of ALU/ALF at 0.25 mg/mL. The light blue box represents the data for pretreatments of the larger combination of PLE at 0.25 mg/mL and ALU/ALF at 0.25 mg/mL, reaching a final concentration of 0.5 mg/mL. For the sake of comparison, the green boxes represent two simulations of the theoretical addition of PLE (0.25 mg/mL) and ALU/ALF (0.25 mg/mL) datasets. The light green box represents ALU/ALF (0.25 mg/mL) data points plus the average value of PLE (0.25 mg/mL). The dark green box represents PLE (0.25 mg/mL) data points plus the average value of ALU/ALF (0.25 mg/mL). The survival of non-treated samples was on average 57.49%. UVB irradiation dose = 0.8 J/cm2. All treatments have equal variances (Leven’s test). All treatments induced statistically significant photoprotection over the control (one-way ANOVA multiple comparisons, post hoc Tukey). The letters above the boxes represent different statistical groups (one-way ANOVA multiple comparisons, post hoc Tukey). *n* = 12 independent wells of cells/group.

We clearly detected PLE-conferred photoprotection in our experimental model (Figure 4, and statistical analysis in Table S5, one-way ANOVA multiple comparisons, post hoc Tukey). At the same concentration, the specific combination of ALU/ALF provided statistically greater photoprotective activity. However, the greatest photoprotective effect was observed when combining ALU/ALF and PLE (50:50) (Figure 4, and statistical analysis in Table S5, one-way ANOVA multiple comparisons, post hoc Tukey). Inquiring on the nature of the observed increase in photoprotection, Figure 4 includes theoretical additions of PLE and ALU/ALF independent results. As no statistical differences were observed between these sums and the real values obtained for PLE/ALU/ALF, it would appear that combining all these extracts provides complementary photoprotective activity (Figure 4, and statistical analysis in Table S5, one-way ANOVA multiple comparisons, post hoc Tukey).

These results show the good performance of ALU/ALF in larger photoprotective mixtures and support the use of PLE and ALU/ALF together in future photoprotective formulations.

## 3. Discussion

The detrimental effects of excessive solar radiation on skin have been well established [3]. This, together with increasing knowledge in the field of photobiology, has triggered a strong interest in the development of prophylactic regimens usually combining different measures [7]. Consequently, there is an active search for new photoprotective ingredients, and natural sources are considered to be the most promising and better accepted by consumers [18,19]. Aiming to identify unexplored natural sources of photoprotective agents, we investigated the properties of two different extracts of *Aspalathus linearis*, which leads us to propose a combination of both extracts as a plausible new photoprotective asset, opening doors for its future inclusion in practical photoprotective protocols.

The first challenge was to develop a simplified cellular model that allowed us to measure the photoprotection conferred by the different extracts. Previous work on the skin chemopreventive properties of *A. linearis* extracts provided comprehensive data in different cellular models. Focusing on skin cancer processes, the studies reported reductions in cell viability when using premalignant human epidermal keratinocytes, such as HaCaT, pretreated with *A. linearis* extracts [56]. The phenotype seems to be related to an induction of apoptosis modulated by pro-inflammatory signals [47] and increased in UV-irradiated cells [46]. This led to the idea that *A. linearis* extracts specifically target DNA-damaged cells, activating their removal by apoptosis. This aforementioned chemopreventive mode of action is proposed to underlie *A. linearis*’ effect in hampering skin tumor development, observed in mice some years before [48]. However, this also pointed to cell viability reduction under UV irradiation in those cellular models, which could difficult its use as a non-pathological model. Conversely, moving the focus to a plausible photoprotective effect, Akinfewa et al. observed, in 2021, how *A. linearis*. extracts are able to limit the UV-induced ROS production, and consequently increase cell viability after UV radiation [32]. Lastly, Keet et al., in 2024, observed how in the same UV-irradiated HaCaT cells, *A. linearis’* antioxidant and anti-inflammatory activity improved the management of UV-induced ROS without a direct effect on cellular viability [31]. Considering our interest in the photoprotective properties of *A. linearis* extracts, we developed a simplified model in normal fibroblasts (NHDF) which avoided the pro-apoptotic effect described in premalignant cells and allowed us to measure cell viability’s high throughput.

We used this experimental setup to characterize the photoprotective effect of unfermented and fermented extracts of *Aspalathus linearis* (ALU and ALF, respectively). We first reproduced the protective effect previously reported with ALU [31,32], validating the model in the absence of cytotoxicity. Some bioactivities associated with *A. linearis* extracts had been previously attributed to specific metabolites, such as three characteristic dihydrochalcones (linearthin, aspalathin, and nothofagin), with an important antioxidant capacity [32,34,56]. As the quantity of these metabolites significantly decreases during fermentation [36,39], it is generally assumed that ALF is less active than ALU [44] and, in fact, this is the case for some of its bioactivities [44]. Surprisingly, in our experimental model, ALF displayed greater photoprotective properties than ALU, and this effect did not correlate with the amount of characteristic metabolites present in the aqueous extract used nor with the in vitro measures of antioxidant activity. Plausible explanations for these results could be related to the concentration ranges of the characteristic metabolites. Actually, Akinfewa et al. reported, in 2021, an increase in cell viability when low concentrations of ALU or the metabolites of interest were used [32]. In that work, the authors used organic ALU extractions and purified samples, employing much higher concentrations than the ones present in aqueous extractions, such as the ones used in this work or in the work presented by Keet et al. in 2024 [31]. It is therefore possible that low concentrations of those compounds could confer cytoprotective effects, and once the concentrations reach certain thresholds, they could induce the pro-apoptotic effects observed in other studies, masking the phenotype on cell viability assays. Another possibility is that the metabolites conferring the photoprotection observed in our model were not the ones considered in previous studies. Indeed, for our characterization, we especially focused on the compounds previously associated with some of the bioactivities reported [61]; however, taking into account the results for ALF, compounds increasing their concentration during fermentation may have a stronger contribution to the photoprotective effect than the ones analyzed. In the future, it would be interesting to explore the photoprotective capacity of, e.g., C-Glucosyl eriodyctiol isomers that have been observed to increase during fermentation [41]. Indeed, eriodyctiol has been reported to confer UVA photoprotection in human skin cells [62], and C-Glucosyl flavones are known to play a role in plant photoprotection [63]. Other approaches could also contemplate performing more detailed metabolic studies [64], or even more interestingly, having in mind the results obtained with the combination ALU/ALF, addressing the interaction between the different metabolites in order to determine their contribution to the photoprotective properties of the extracts.

Beyond the considerations regarding the role of specific metabolites, the phenotype observed in ALF and the specific ALU/ALF combination did not correlate with the antioxidant capacity of the samples measured in vitro (FRAP analysis), which had been previously attributed to *A. linearis* properties [59]. In this respect, it could be important to differentiate the physicochemical antioxidant capacity from the antioxidant capacity induced in the cell. In the case of a *Polypodium leucotomos* extract (PLE, commercialized as Fernblock^®^), a well-known photoprotectant, the beneficial effects have been more associated with an induction of the endogenous antioxidant systems in the cells, such as NRF2 protein [65,66], than with the physicochemical antioxidant capacity [55]. This could be the case for *A. linearis* extracts, which have also been demonstrated to induce endogenous antioxidant cell systems, including NRF2 [67]. Additionally, even though ALF possesses lower in vitro antioxidant capacity, it is able to induce total plasma antioxidant capacity in vivo to a similar or even higher extent than ALU [42]. Moreover, we used pretreatments with the extracts before subjecting the cells to the irradiation stress (washing the treatments before irradiating), similar to the model used by Keet et al. 2024, where the group reported inductions in the cellular antioxidant systems even in the absence of radiation, and observed how the extracts counteract the subsequent effect of UV radiation [31]. Thus, it could be possible that *A. linearis* extracts prime the endogenous protective cell systems to minimize the impact of UV damage, resulting in the increases in cell survival observed in our model.

Interestingly, from a translational science perspective, we identified a specific combination of ALU/ALF with increased photoprotection and provided a complementary effect to Fernblock^®^. It is planned that this combination of ALU/ALF and Fernblock^®^ will be commercialized as Aspa-Fernblock^®^. Synergic properties of natural extracts have been reported [57,58], and they could be particularly relevant when integrating this new technology in practical photoprotection measures as they usually consist of complex formulations with benefits emerging for such combinations [14,60]. The case of Aspa-Fernblock^®^ could be of special interest given the many reported benefits of Fernblock^®^ in both topical and oral photoprotective strategies [55]. On one hand, in the case of sunscreens, there is evidence that some natural antioxidant compounds and Fernblock^®^ can improve light-filtering properties and photostability [22,27,28]. On the other, for alternative systemic photoprotective approaches, Fernblock^®^ represents one of the most widely studied ingredients, with beneficial results supported by clinical trials in healthy individuals and vulnerable populations [17,55]. Thus, considering the results obtained in this work, future research will reasonably include the characterization of the optical properties of Aspa-Fernblock^®^, its additional contribution in final sunscreen formulations, and, possibly, its efficacy as an oral photoprotector in clinical trials.

In conclusion, aiming to expand the current array of natural photoprotection agents, we analyzed the properties of two different extracts of *Aspalathus linearis*, and presented a new photoprotective technology derived from a specific combination of both extracts. This technology showed enhanced photoprotective activities vs. each extract on its own, and a complementary effect when combined with Fernblock^®^. These results pave the way for subsequent research investigating the beneficial properties of *A. linearis*, but also open doors for exploring its potential for developing more natural and effective photoprotection strategies.

## 4. Materials and Methods

### 4.1. Biological Material and Extract Preparation

#### 4.1.1. Plant Extracts

The *Polypodium leucotomos* solid extract used in the experiments was provided by Industrial Farmaceutica Cantabria, S.A., Madrid, Spain. The unfermented and fermented *Aspalathus linearis* solid extracts used were bought from Rooibos Ltd. (Clanwilliam, Western Cape, South Africa).

#### 4.1.2. Extract Preparation

Extract preparation for photoprotection and FRAP assays was carried out as follows: the same weight of both extracts was measured, dissolved in DMEM low-glucose (L1P04-01159, Panbiotech, Aidenbach, Germany) supplemented with L-Glutamine 200 mM (25-0005-CI, Corning Inc., Corning, NY, USA) and Penicillin–Streptomycin (10.000 IU-10.000 μg/mL, 30-002-CI, Corning Inc., Corning, NY, USA), and stirred at 37 °C for 30 min. The treatments were performed with the samples dissolved in supplemented DMEM low-glucose medium without phenol red to avoid interference with the treatments/irradiation. The samples were filtered through a PVDF 0.45 µm membrane. For sample combinations, the desired final concentration (total ALU/ALF) was achieved by adding the solid weight for each of the extracts and considering the total solid weight/volume (μg/mL). The combination of the extracts was performed in liquid solutions under sterile conditions.

### 4.2. Photoprotection Assay

#### 4.2.1. Cell Culture

Normal Human Dermal Fibroblasts (NHDF, K3CC-2511, Lonza, Basel, Switzerland) were cultured in high-glucose Dulbecco’s modified Eagle’s medium (DMEM, D5671, Sigma Aldrich, San Louis, MI, USA) supplemented with fetal bovine serum 10% (FBS, 35-015-CF, Corning Inc., Corning, New York, NY, USA), L-Glutamine 200 mM (25-0005-CI, Corning Inc., Corning, New York, NY, USA), and Penicillin–Streptomycin (10.000 IU-10.000 μg/mL, 30-002-CI, Corning Inc., Corning, New York, NY, USA). Supplemented high-glucose DMEM with phenol red was employed in all steps for growing cells or when the cells were left to recover after treatment/UVB. The cells were incubated in an atmosphere of 5% CO_2_ at 37 °C (BINDER CB-S E7 180). When they reached 80% confluence, the cells were washed with Hank’s Balanced Salt solution (H6648, Sigma Aldrich, San Louis, MI, USA) and harvested using Trypsin-EDTA 0.5% (MS02241019, LINUS, Witten, Germany). The cells were considered suitable for assays from passages 6 to 18. Cell concentration was adjusted to 6 × 10^4^ cells/μL for every assay performed. As preparation for each assay, the cells were cultured in 96-well plates Nunc (2029-03, Thermo Fisher, Waltham, MA, USA) for 24 h at 5% CO_2_ and 37 °C. For cell counting, a Neubauer Chamber (717805, Blaubrand, Wertheim, Germany) was used.

#### 4.2.2. Cell Viability Assessment

Crystal violet was employed to assess cell viability [68]. The cells were washed with 1X PBS twice to remove the dead (detached) cells. Surviving (attached) cells were fixed with 1X PBS + 25% glutaraldehyde for 15 min at RT and thoroughly washed with water. Then, 0.5% crystal violet (C0775, Sigma Aldrich, San Louis, MI, USA) [68,69] in 20% absolute ethanol (131085.1214, PanReac AppliChem, Barcelona, Spain) was added and incubated during shaking (200 rpm orbital shaker RSLab LJN005) for 20 min. The plates were then thoroughly washed with water several times and left to dry for 2–3 h. Following this, 96% ethanol was added for resuspension under shaking for 20 min. Finally, 540 nm OD data collection was performed using a BMG Labtech, FLUOstar Optima (Offenburg, Germany).

#### 4.2.3. Irradiation Test

Every new thawed cell line underwent an irradiation assay to determine the precise irradiation intensity at which cell survival rates (of non-treated samples) were between 40 and 60%. The cells were cultured (4.2.1) by sowing at least one plate per intensity to be tested. The cells were washed twice with DMEM low-glucose supplemented with L-Glutamine 200 mM, Penicillin–Streptomycin (10.000 IU–10.000 μg/mL), and incubated for 24 h under the same conditions. At 48 h and after washing them twice with Hank’s Balanced Salt Solution, the plates were irradiated or not with a broadband UVB lamp (Bio-Link -BLX- Vilber, Marne La Valleé, France). Afterward, they were washed with Hank’s Solution and allowed to rest for 24 h at 5% CO_2_ and 37 °C in DMEM high-glucose supplemented with fetal bovine serum 10%, L-Glutamine 200 mM, and Penicillin-Streptomycin (10.000 IU-10.000 μg/mL). At 72 h, the cells were analyzed using the cell viability assessment (4.2.2).

#### 4.2.4. Cytotoxicity Test

Cytotoxicity was analyzed based on measurements of cell growth (4.2.2 Cell viability assessment) relative to non-treated cells and expressed as percentages (growth in non-treated cells considered 100%). Both extracts, ALU and ALF, were tested at different concentrations between 10 and 1000 μg/mL. The cells were cultured (4.2.1), treated with the extracts, and analyzed using the cell viability assessment (4.2.2).

#### 4.2.5. Photoprotection Assessment (Figure S1)

- 0 h. The cell suspension was centrifuged in supplemented high-glucose DMEM for 10 min at 1500 rpm, 240× *g* at room temperature. The pellet was resuspended in supplemented high-glucose DMEM. The cells were cultured as described in Section 4.2.1. Perimetrical wells were assigned as blank controls (supplemented high-glucose DMEM and no cells). At least two plates were seeded per assay condition.
- 24 h. All the plates were washed with supplemented low-glucose DMEM. At least 12 wells were used on each plate as non-treated control fibroblasts. Then, 200 μL of low-glucose supplemented DMEM was added to non-treated fibroblasts and blanks (perimetral wells). Following this, 200 μL of the treatments (prepared in low-glucose supplemented DMEM, as described in Section 4.1.2) were applied to the corresponding wells. The cells were incubated for 24 h at 5% CO_2_ and 37 °C.
- 48 h. The cells were washed twice with Hank’s Balanced Salt solution, and 100 μL of fresh Hank’s was added. Half of the plates were irradiated with a broadband UVB lamp. The other half of the plates in each assay represented the non-irradiated control. After UVB exposure (or control), the medium was discarded. The cells were rewashed with Hank’s Balanced Salt solution and kept in 200 μL high-glucose supplemented DMEM for the following 24 h at 5% CO_2_ and 37 °C.
- 72 h. The cells were analyzed using cell viability assessment (Section 4.2.2).

#### 4.2.6. Data Analysis

After the photoprotection assessment, OD data were first processed by removing the background signal obtained from the blank samples (without fibroblasts). Then, the survival rates expressed in percentages were calculated from UV-irradiated fibroblasts relative to non-irradiated fibroblasts for each group (assuming non-irradiated fibroblast growth quantification as 100%) in order to avoid bias due to different cell growth under the treatments regardless of irradiation.

$$A:\;\%\;Survival\;control\;{UV}^{+}\;relative\;to\;control\;growth\;in\;{UV}^{-}$$

$$A=\frac{\left(Absorbance\;of\;each\;control\;sample\;{UV}^{+}\;-\;blank\right)\;\times \;100}{\bar{x}\;controls\;{UV}^{-}}$$

$$B:\;\%\;Survival\;{UV}^{+}\;of\;a\;treatment\;relative\;to\;its\;growth\;in\;{UV}^{-}$$

$$B=\frac{\left(Absorbance\;of\;each\;{UV}^{+}\;treated\;sample\;-\;blank\right)\;\times \;100}{\bar{x}\;treatment\;{UV}^{-}}$$

The survival of the control non-treated UV-irradiated samples was considered “no induced-photoprotection” (A). The increase in the survival rates of the treated groups (B) vs. the control non-treated group (A) is thus expressed as “Photoprotection Relative to Control (PT)”.

$$Relative\;Photoprotection\;Over\;Control\;\left(PT\right)\;=\;Survival\;{UV}^{+}\;-\;Survival\;{UV}^{+}\;of\;the\;controls$$

PT=B−A

Hence, the photoprotection rates shown for each treatment represent the average increase in survival rates (of UV-irradiated cells) relative to non-treated samples.

### 4.3. HPLC Analysis

#### 4.3.1. Chemicals and Reagents

All the chemicals used were of analytical grade. Acetonitrile (CAS No. 75-05-8, purity (GC) 99.9%) and Dimethyl sulfoxide (DMSO, CAS No. 67-68-5, purity (GC) 99.9%) were provided by Merck (Darmstadt, Germany). Glacial acetic acid (CAS No. 64-19-7, purity (GC) ≥99.7%) was obtained from Panreac AppliChem (Barcelona, Spain). Ascorbic acid (CAS No. 50-81-7, purity (titration by iodine) 99%) was provided by Sigma Aldrich (Darmstadt, Germany). The following analytical standards were used to identify and quantify polyphenols: Aspalathin (CAS No. 6027-43-6, purity (HPLC) 90.0%, PhytoLab GmbH & Co. KG, Vestenbergsgreuth, Germany), Isoorientin (CAS No. 4261-42-1, purity (HPLC) 97.5%, Biopurify Phytochemicals, Chengdu, China), Luteolin (CAS No. 491-70-3, purity (HPLC) 98.1%, Biopurify Phytochemicals, Chengdu, China), Quercetin (CAS No. 117-39-5, purity (HPLC) 96.0%, Glentham^®^ Life Sciences GmbH, Planegg, Germany), Nothofagin (CAS No. 11023-94-2, purity (HPLC) 99.21%, Targetmol Pamplona, Spain), Orientin (CAS number: 28608-75-5, purity (HPLC) 99.35%, Targetmol, Pamplona, Spain), Vitexin (CAS number: 3681-93-4, purity (HPLC) 98.5%, Biopurify Phytochemicals, Chengdu, China), Isovitexin (CAS No. 38953-85-4, (HPLC) 96.5%, Biopurify Phytochemicals, Chengdu, China), Rutin (CAS No. 153-18-4 (HPLC) 96.0%, Cymit Química S.L., Pamplona, Spain) and Hyperoside (CAS No. 482-36-0 (HPLC), 97.3% Biopurify Phytochemicals, Chengdu, China).

#### 4.3.2. Preparation of Standards, Calibration Curves, and Solutions

Solvent A: 2% *v*/*v* acetic acid. Briefly, 20 mL of glacial acetic acid was transferred to a 1000 mL volumetric flask with purified water. Before use, the solution was filtered through 0.22 µm and degassed. Solvent B: acetonitrile 100%. Before use, the solution was filtered through 0.22 µm and degassed. Then, a 10% ascorbic acid solution was prepared by transferring 1 g of ascorbic acid, dissolved and diluted with purified water to a 10 mL volumetric flask.

Individual stock reference solutions of each phenolic compound were prepared in DMSO by weighing 5 mg of each analyte into 5 mL volumetric flask and sonicating these for 5 min. All standard solutions were stored protected from light and air at −20 °C in topaz HPLC vials. The UV spectrum and retention time of individual compounds are shown in Figure S7.

For the quantification of phenolic compounds in unfermented and fermented *Aspalathus linearis,* solid extracts were calculated by preparing a calibration curve of mass. Three-point calibration curves with phenolic compounds were prepared by serial dilution to contain 65.0 µg/mL, 30.0 µg/mL and 0.5 µg/mL Aspalathin; and 15.0 µg/mL, 7.5 µg/mL and 0.2 µL/mL Isoorientin, Luteolin, Quercetin, Nothofagin, Orientin, Vitexin, Isovitexin, Rutin and Hyperoside. Calibration solutions were prepared by transferring the indicated volume of stock reference solution into a 5 mL volumetric flask, adding 25 µL of 10% ascorbic acid solution, diluting with purified water, and sonicating for 2 min. All standards were stored protected from light and air at 4–8 °C in topaz HPLC vials.

#### 4.3.3. Preparation of Sample Solutions of ALU and ALF for Identification and Quantification of Phenolic Compounds by HPLC

For the identification by retention time and UV spectrum, and quantification of phenolic compound by area, 100 mg of unfermented and fermented *Aspalathus linearis* solid extracts was transferred to 100 mL volumetric flasks. Then, 500 µL of 10% ascorbic acid solution was added. The samples were sonicated for 10 min and diluted to volume with solvent A (2% *v*/*v* acetic acid)/solvent B (acetonitrile) at a ratio of 90:10. The samples were then filtered through PVDF membranes of 0.22 µm to a HPLC vial. Chromatographic profiles of ALU and ALF are shown in Figures S8 and S9, respectively.

To confirm the identity of the compounds by retention time and UV spectrum, the indicated volume of the stock reference solution for each compound (100 µL of aspalathin; 10.0 µL of nothofagin, 10.0 µL of orientin; 15.0 µL of isoorientin; 5.0 µL of vitexin; 15.0 µL isovitexin; 2.5 µL of luteolin; 7.5 µL rutin; 2.5 µL quercetin; 2.5 µL of hyperoside) was transferred into 5 mL volumetric flask, and 25 µL of 10% ascorbic acid solution was added. The sample was then diluted to volume with *Aspalathus* linearis extracts, sonicated and filtered through PVDF membranes of 0.22 µm to a HPLC vial.

#### 4.3.4. Chromatographic Conditions

The HPLC method employed in this study with some modifications was described by Beelders T. et al. [61]. Phenolic analysis was performed by HPLC with a DAD detector (Agilent 1260 Infinity II, Madrid, Spain) with an Agilent Zorbax SB-C18, 1.8 µm, 100 × 4.6 mm column (Agilent, Madrid, Spain). The mobile phase consisted of a gradient elution using the proportions of solvent A (2% *v*/*v* acetic acid in MiliQ water) to solvent B (acetonitrile) as follows: initial 90% A; 0–19 min, 85.2% A; 19–34 min, 63.2% A; 34–37 min, 0% A, 37–45 min, 90% A, 42–50 min, with a flow rate of 1 mL/min and an injection volume of 50 µL of samples and standards. The column temperature was maintained at 37 °C throughout the analysis and the spectra were acquired in the 200–700 nm range. Chromatograms were plotted at 288 and 350 nm. Using these chromatographic conditions, the retention time of compounds was confirmed by injection of the corresponding standard separately.

The HPLC method was performed in accordance with the ICH guidelines, including specificity, linearity, LOD, LOQ, precision, and accuracy [70].

### 4.4. Ferric Reducing Antioxidant Assay (FRAP)

Treatments were prepared as in Section 4.1.2. Each sample was placed in a Nunc 96-well plate and diluted in FRAP reactive (TPTZ -2,4,6-trispyridil-s-triazine- 10 mM (93285, Merck, Darmstadt, Germany), FeCl_3_ 20mM (701122, Merck, Darmstadt, Germany), and acetate buffer (SO00300250, Scharlab, Barcelona, Spain) following 10:1:1, 37 °C). Then, 0.2 mM of Trolox C (6-hidroxy-2,5,7,8-tetramethylchromane-2-carboxylic acid; 238813, Merck, Darmstadt, Germany) prepared in ethanol was used as a positive control. For FRAP quantification, OD reading (BMG Labtech, FLUOstar Óptima) set up at 593 nm was performed. OD readings for Trolox were used to obtain the Trolox linear equation (Figure S6a). For the treatments of interest, relative values to Trolox (μg of Trolox) were calculated by interpolating the obtained absorbances in the Trolox linear equation.

### 4.5. Statistics

For the irradiation test, the data were analyzed (linear regression analysis) using Minitab Statistical Software 22.0.1 (Minitab LLC, State College, PA, USA). For the rest of the statistical analysis presented here (one or two-way ANOVA), SPSS Software V23.0 (IBM, Armonk, NY, USA) was employed.

## 5. Patents

The specific combination of ALU and ALF presented here is currently patent-pending by Cantabria Labs.

## Figures and Tables

**Table 1:** Quantification of ALU and ALF metabolites by HPLC.

Compound	* %w/w ± Standard Deviation (*SD*)	
	ALU	ALF
Isoorientin	0.93 ± 0.02	0.50 ± 0.01
Orientin	1.03 ± 0.01	0.26 ± 0.01
Aspalathin	5.00 ± 0.02	0.08 ± 0.00
Vitexin	0.19 ± 0.00	0.04 ± 0.00
Hyperoside	0.02 ± 0.00	<0.01 ± 0.00
Rutin	0.05 ± 0.01	0.01 ± 0.00
Isovitexin	0.19 ± 0.00	0.11 ± 0.01
Nothofagin	0.85 ± 0.00	0.04 ± 0.00
Quercetin	0.59 ± 0.03	0.20 ± 0.00
Luteolin	0.05 ± 0.00	0.27 ± 0.02

* Percentage weight/weight. Two replicates of each sample were carried out. Data were expressed as means ± standard deviation (*SD*).

## Data Availability

The main part of the original contributions presented in this study are included in the article/Supplementary Material. However, further inquiries about raw data can be directed to the corresponding authors.

## Supplementary Materials

The following supporting information can be downloaded at: https://www.mdpi.com/article/10.3390/ijms26052330/s1.
