# Supplementary material for: Exploring the In Vitro Photoprotective Effect of a Combination of Aspalathus linearis Natural Extracts: First Steps in Developing New Technologies for Photoprotection Strategies
Source: Int J Mol Sci. 2025 Mar 5;26(5):2330. doi: 10.3390/ijms26052330 (PMC11900052; doi:10.3390/ijms26052330)
Supplement: Supplementary file 1 [file ijms-26-02330-s001.zip › ijms-3435459-supplementary.pdf]

Supplementary Materials:

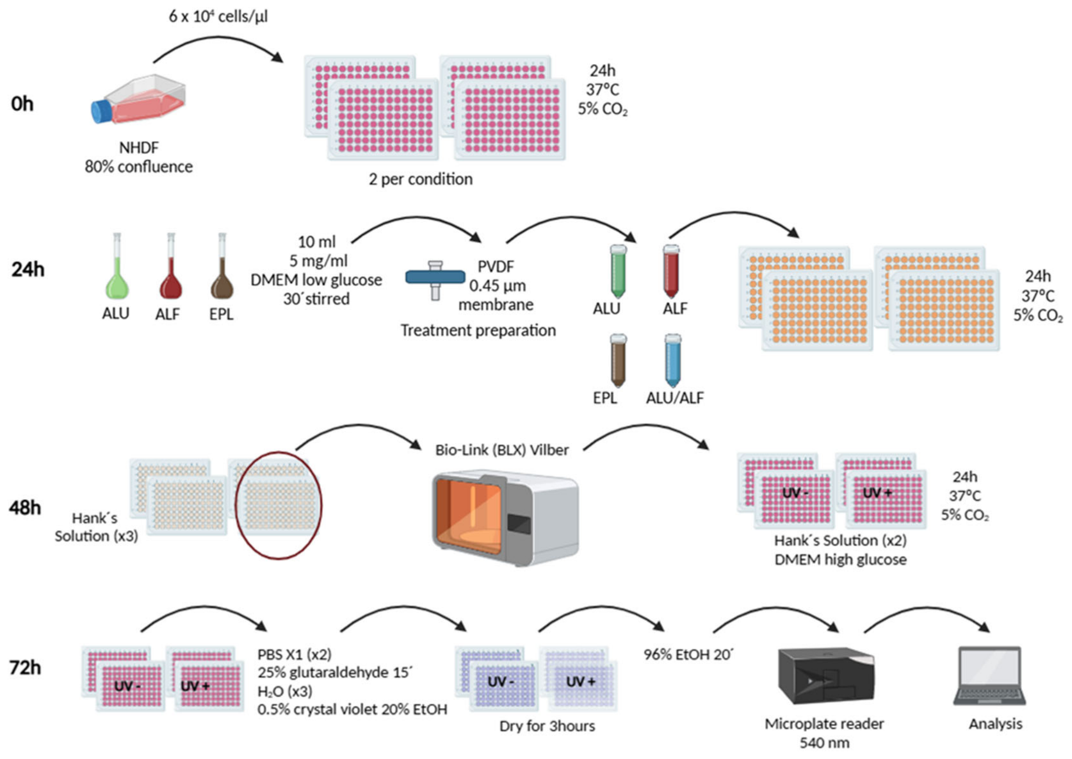

Figure S1. Experimental setup for photoprotection assay. The assay is performed over 4 consecutive days. The image was generated using Biorender (Biorender.com).

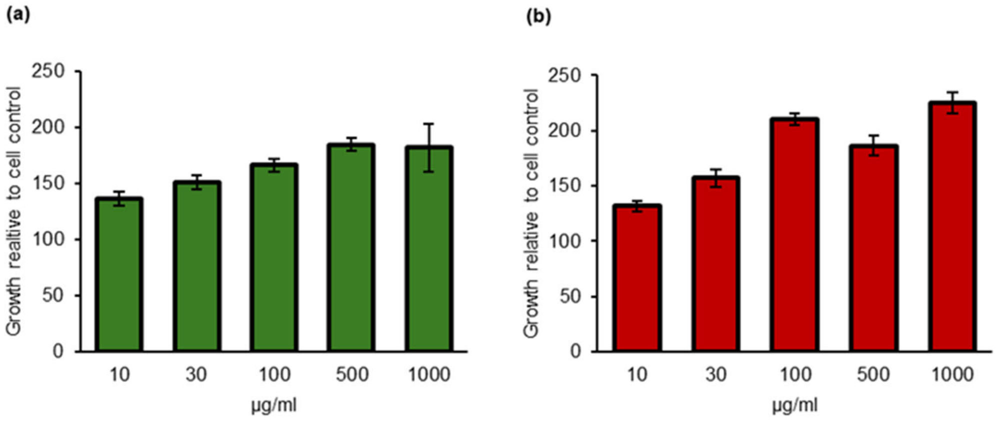

Figure S2. Cytotoxicity assay. Cytotoxicity for ALU and ALF was analyzed based on cell growth relative to non-treated cells and expressed as percentages (growth in non-treated cells considered 100%). Five different concentrations (10-1000µg/ml) were tested for each extract. (**a**) ALU. (**b**) ALF. The Y-axis represents average values. n ≥ 50. Error bars represent SEM (standard error of the mean).

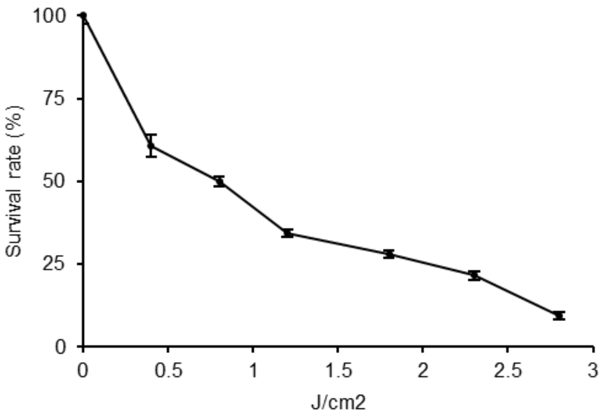

(**a**)

Regression Equation

% survival = 79,96 - 27,52 J/cm2

Coefficients

Term	Coef	SE Coef	T-Value	P-Value	VIF
Constant	79,96	8,08	9,90	0,000	
J/cm2	-27,52	4,95	-5,56	0,003	1,00

Model Summary

S	R-sq	R-sq(adj)	R-sq(pred)
12,3862	86,06%	83,27%	65,80%

Analysis of Variance

Source	DF	Adj SS	Adj MS	F-Value	P-Value
Regression	1	4735,5	4735,5	30,87	0,003
J/cm2	1	4735,5	4735,5	30,87	0,003
Error	5	767,1	153,4		
Total	6	5502,6			

(**b**)

Figure S3. Irradiation test. (**a**) NHDF survival rates after UV irradiation were tested between 0.4 and 2.8 J/cm^2. The Y-axis represents average values. n ≥ 15 for each data point at the six different irradiation intensities. Error bars represent SEM (standard error of the mean). (**b**) Statistical analysis by Regression Equation: %Survival = 79.95 -27.52 J/cm^2).

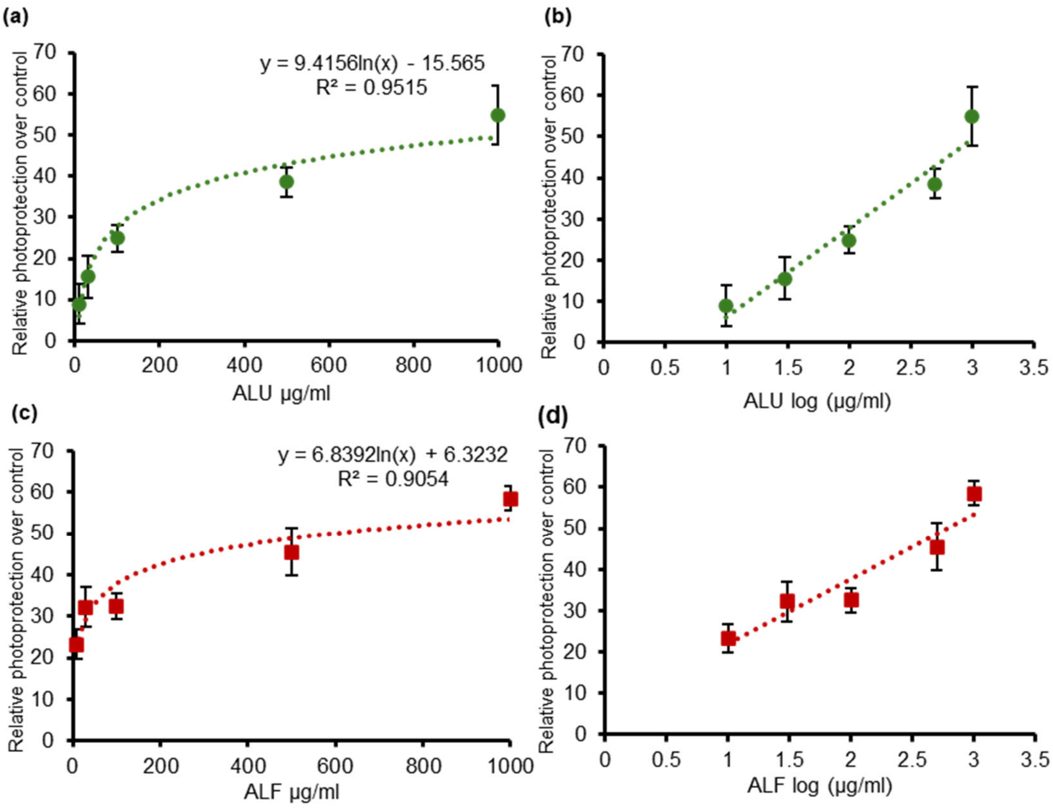

Figure S4. Photoprotective effect of ALU and ALF aqueous extracts. (**a**,**b**) Photoprotection induced by ALU treatments; (**c**,**d**) Photoprotection induced by ALF treatments. The X-axis represents the concentration of the extracts used in the treatments, expressed in linear scale in graphs (**a**,**c**); and in a logarithmic scale in (**b**,**d**). The Y-axis represents the average values of relative photoprotection measures. Survival of non-treated samples was 54.75%. UVB irradiation dose = 0.8 J/cm2. The different extracts were tested at 10, 30, 100, 500, and 1000 µg/ml. For all data points n ≥ 4 independent wells of cells. Error bars represent the standard error of the mean (*SEM*). Data trendlines are drawn.

Table S1. Statistical two-way ANOVA analysis corresponding to Figure 1.

Univariate Analysis of Variance

Warnings

Post hoc tests are not performed for Treatment because there are fewer than three groups.

Between-Subjects Factors

		Value Label	N
Treatment	1	ALF	26
	2	ALU	23
Concentration	10		11
	30		10
	100		9
	500		10
	1000		9

Tests of Between-Subjects Effects

Dependent Variable: Relative -Photoprotection over control

Source	Type III Sum of Squares	df	Mean Square	F	Sig.
Corrected Model	11954.199	9	1328.244	9.889	.000
Intercept	53759.741	1	53759.741	400.270	.000
Treatment	1155.780	1	1155.780	8.605	.006
Concentration	9873.095	4	2468.274	18.378	.000
Treatment * Concentration	287.353	4	71.838	.535	.711
Error	5238.038	39	134.309		
Total	70054.308	49			
Corrected Total	17192.237	48			

Estimated Marginal Means

Treatment

Dependent Variable: Relative -photoprotection over control

Treatment	Mean	Std. Error	95% Confidence Interval	
			Lower Bound	Upper Bound
ALF	38.373	2.279	33.764	42.983
ALU	28.559	2.449	23.605	33.514

Profile Plots

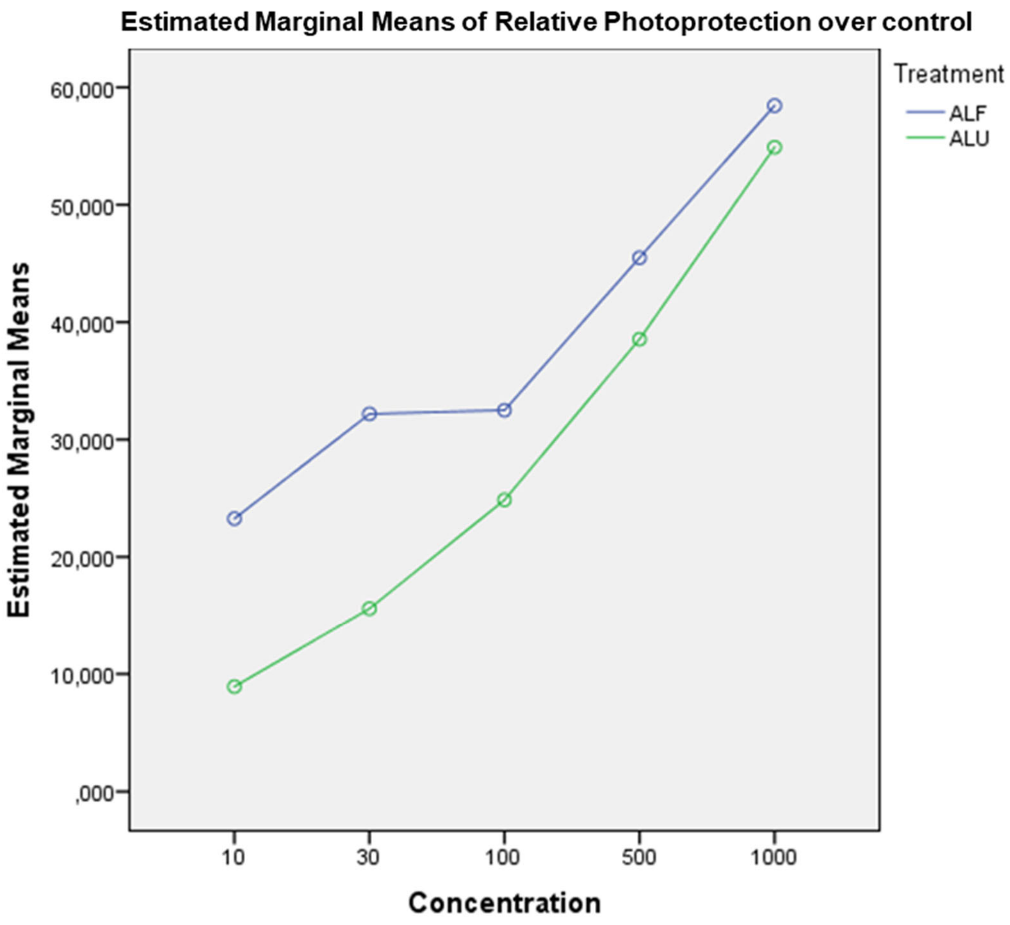

Table S2. Characteristics of calibration curves obtained for HPLC analysis of standards. .

Standard	Concentration Range	[a]Regression Equation	Correlation Coefficient, r
Isoorientin	0.2 – 15.0 μg/ml	y = 142.18 – 8.40	1.0000
Orientin		y = 135.02 – 6.70	1.0000
Aspalathin	0.5 – 65.0 μg/ml	y = 113.40 + 12.03	1.0000
Vitexin	0.2 – 15.0 μg/ml	y = 113.19 – 3.50	1.0000
Hyperoside		y = 103.02 – 3.62	1.0000
Rutin		y = 61.06 + 0.48	1.0000
Isovitexine		y = 113.00 – 4.82	1.0000
Nothofagin		y = 92.17 + 2.26	1.0000
Quercitin		y = 136.29 – 26.09	1.0000
Luteolin		y = 203.70 – 61.65	0.9998

*Number of calibration points, n = 3.

[a] y =analyte response (peak area in mAU) and x = amount of standard (μg/ml)

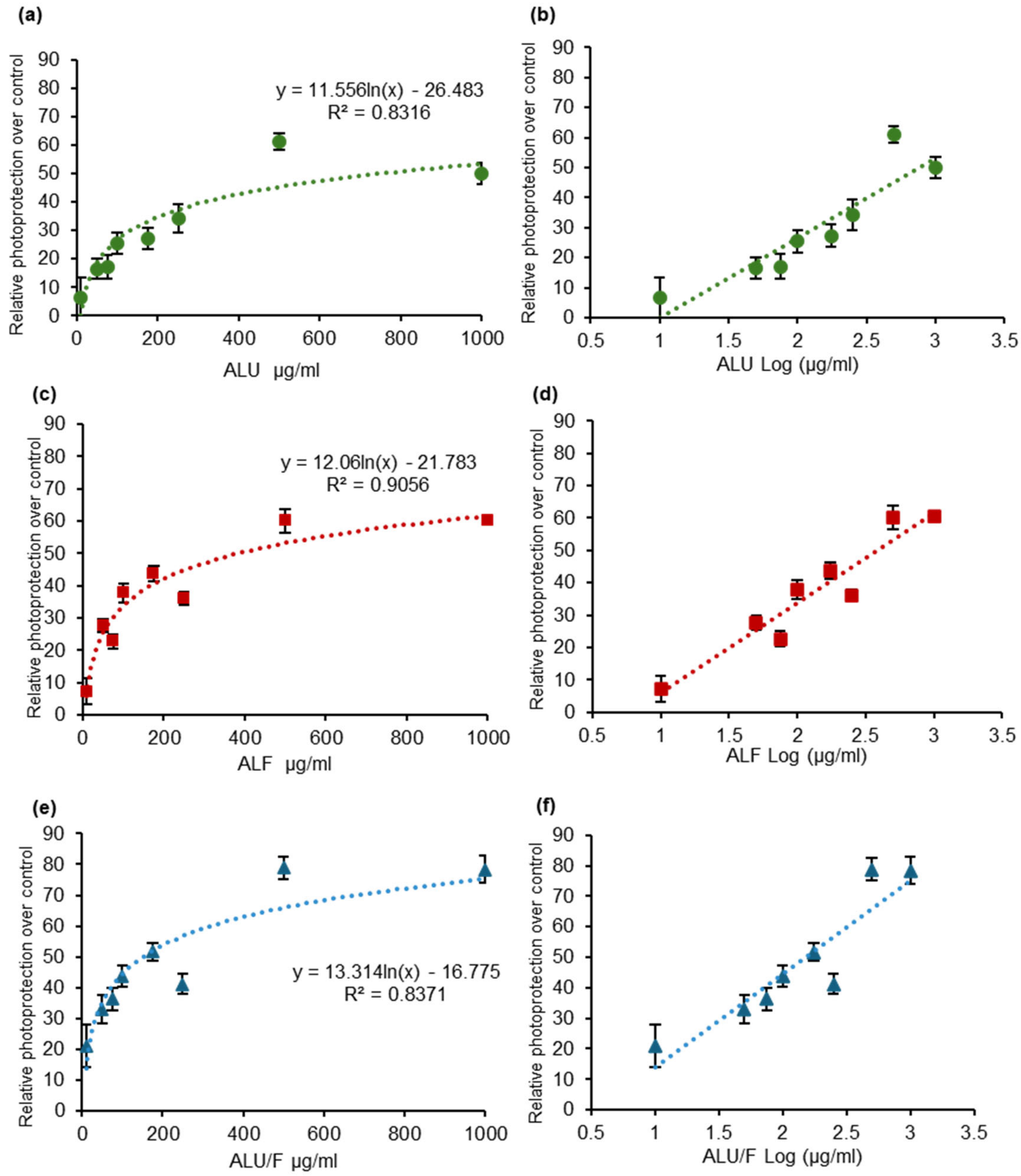

Figure S5. Photoprotective effect of ALU, ALF, and a specific combination ALU/ALF. (**a**,**b**) Photoprotection induced by ALU treatments; (**c**,**d**) Photoprotection induced by ALF treatments; (**e**,**f**) Photoprotection induced by ALU/ALF treatments. The X-axis represents the concentration of the extracts used in the treatments, expressed in linear scale in graphs (**a**,**c**,**e**); and in a logarithmic scale in (**b**,**d**,**f**). The Y-axis represents the average values of relative photoprotection measures shown by the fibroblasts. For all samples $n \geq 4$ independent wells of cells. Error bars represent the standard error of the mean (*SEM*). Data trendlines are drawn.

Table S3. Statistical two-way ANOVA analysis and multiple comparisons corresponding to Figure 2.

Univariate Analysis of Variance

Between-Subjects Factors

		Value Label	N
Treatment	1	ALF	42
	2	ALU	45
	3	ALU/ALF	45
Concentration	10		18
	50		14
	75		17
	100		16
	175		16
	250		17
	500		17
	1000		17

Tests of Between-Subjects Effects

Dependent Variable: Relative Photoprotection over control

Source	Type III Sum of Squares	df	Mean Square	F	Sig.
Corrected Model	51009.419	23	2217.801	25.747	.000
Intercept	189342.713	1	189342.713	2198.167	.000
Treatment	7557.412	2	3778.706	43.869	.000
Concentration	41870.015	7	5981.431	69.441	.000
Treatment * concentration	1395.250	14	99.661	1.157	.319
Error	9302.757	108	86.137		
Total	253733.677	132			
Corrected Total	60312.176	131			

Estimated Marginal Means

Treatment

Dependent Variable: Relative Photoprotection over control

Treatment	Mean	Std. Error	95% Confidence Interval Lower Bound	95% Confidence Interval Upper Bound
ALF	36.926	1.460	34.032	39.820
ALU	29.774	1.389	27.021	32.527
ALU/ALF	48.040	1.389	45.286	50.793

Post hoc

Homogeneous Subsets

Relative photoprotection over control

Tukey HSD

Treatment	N	Subset 1	2	3
ALU	45	29.90059		
ALF	42		37.01119	
ALU/ALF	45			47.84195
Sig.		1.000	1.000	1.000

Profile Plots

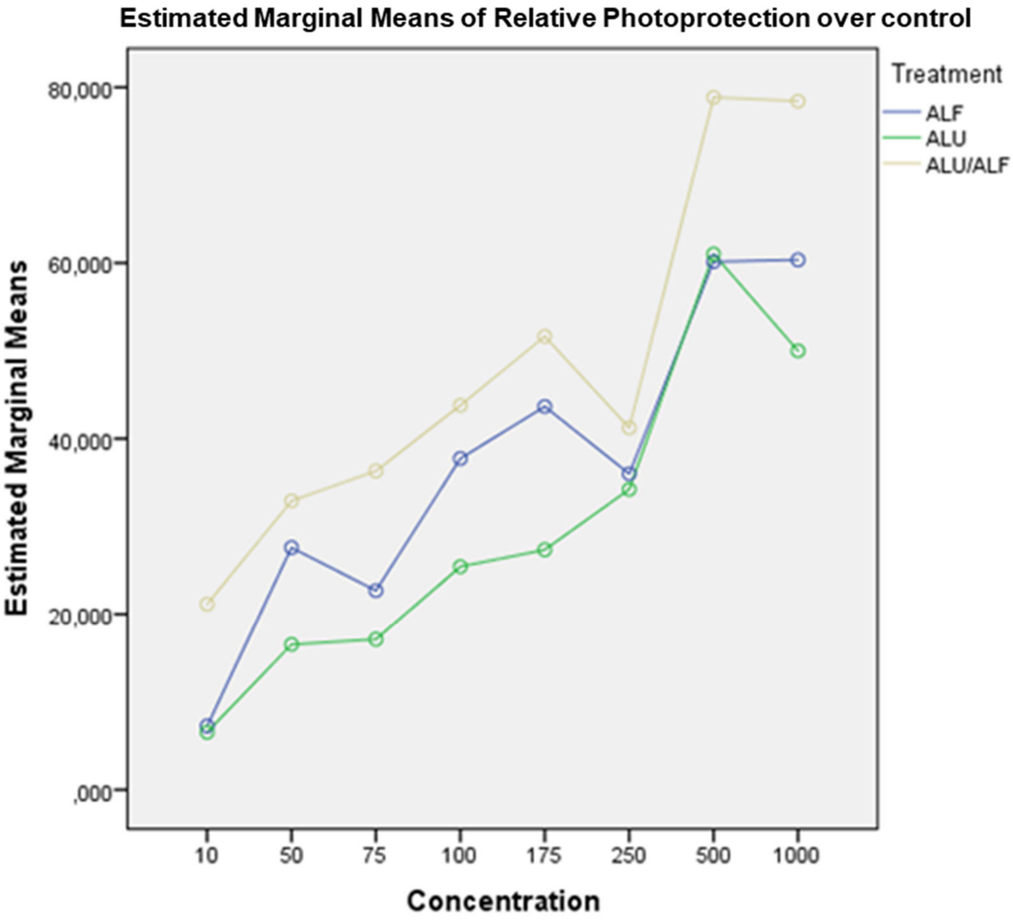

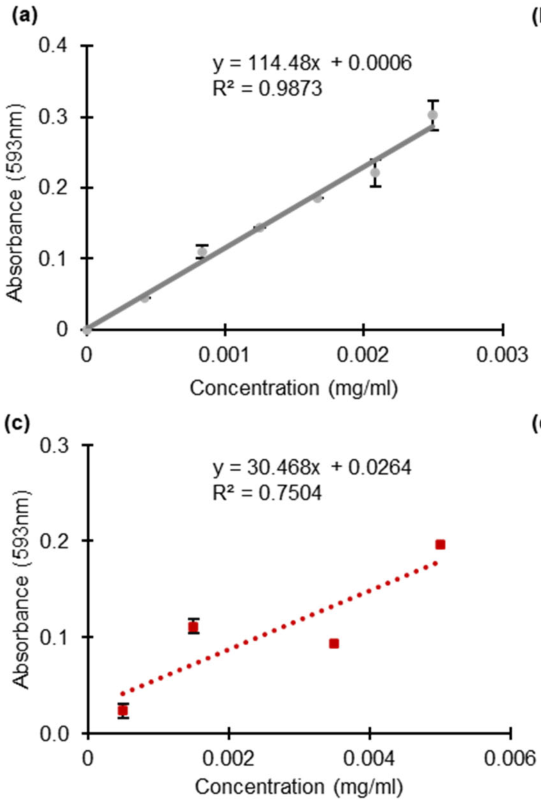

Figure S6. Antioxidant capacity of ALU, ALF, and ALU/ALF analyzed by Ferric Reducing Antioxidant Power (FRAP). (**a**) Trolox is used as an antioxidant of reference (positive control). FRAP signal (absorbance at 593 nm) was used to calculate Trolox linear equation. (**b**–**d**) are FRAP data from ALU, ALF, and ALU/ALF relative to Trolox (mg of Trolox). For all different samples, the X-axis represents the concentrations (μ/ml) at which the extracts were tested. For all data points *n* = 3 independent wells of cells. Error bars represent the standard error of the mean (*SEM*).

Table S4. % of FRAP antioxidant capacities relative to Trolox. Relative values were obtained by interpolating the corresponding concentrations in Trolox linear equation (calculated in Figure S6a). For the samples of interest, absorbance values are expressed as percentages of Trolox for each concentration.

	Relative to $TROLOX_{(1)}$ (%)		
Concentration mg/ml	**ALU**	**ALF**	**ALU/F**
0.0005	55.51	40.95	38.04
0.0015	51.50	38.11	41.22
0.0035	50.52	34.55	39.88
0.005	47.71	34.20	34.09
AVERAGE	51.31	36.95	38.30
SEM ±	1.40	1.38	1.34

(1) Trolox values obtained by interpolations on the calibration curve.
*There is a 27.99% difference in the antioxidant capacity between ALF and ALU.

Table S5. Statistical one-way ANOVA analysis and multiple comparisons corresponding to Figure 4.

Descriptive

Measured Variable

	N	Mean	Std. Desviation	Std. Error	95% Confidence Interval Lower Bound	95% Confidence Interval Upper Bound	Minimum	Maximum
Control	56	-.000000000	9.214666220	1.231361569	-2.46770372	2.467703729	-25.8659561	19.47120312
EPL	14	17.34868115	7.558483441	2.020089671	12.98454274	21.71281956	8.200142462	30.10848511
ALF/ALU	12	78.66799430	8.480193334	2.448020952	73.27993651	84.05605208	66.99063648	94.06325528
EPL + ALF/ALU	12	91.76206549	7.936282492	2.291007417	86.71959217	96.80453882	81.03181214	106.8090874
S. XEPL + ALF/ALU	12	96.01667545	8.480193334	2.448020952	90.62861766	101.4047332	84.33931763	111.4119364
S. X ALF/ALU + EPL	14	96.01667545	7.558483441	2.020089671	91.65253704	100.3808138	86.86813676	108.7764794
Total	120	39.87063179	44.61698762	4.072955095	31.80577391	47.93548967	-25.8659561	111.4119364

Test for homogeneity of variances

Measured Variable

Levene´s	gl1	gl2	Sig.
.195	5	114	.964

ANOVA

Measured Variable

	Sum of squares	gl	Mean Square	F	Sig.
Between Groups	228460.012	5	45692.002	617.871	.000
Within Groups	8430.383	114	73.951		
Total	236890.395	119			

Post hoc

Homogeneous subsests

Measured Variable

HSD Tukey

Treatment	N	Subset for alpha = 0.05			
		1	2	3	4
Control	56	-.000000000			
EPL	14		17.3486811500		
ALF/ALU	12			78.6679943000	
EPL + ALF/ALU	12				91.7620654900
S. XEPL + ALF/ALU	12				96.0166754500
S. X ALF/ALU + EPL	14				96.0166754500
Total		1.000	1.000	1.000	.764

Compound	Retention time	UV spectra
Aspalathin	≈10.0	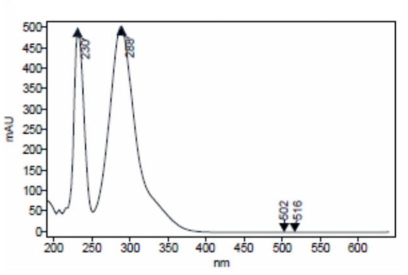
Luteolin	≈27.9	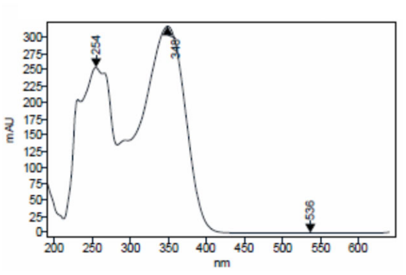
Quercetin	≈27.5	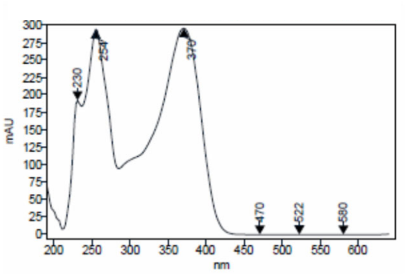
Isoorientin	≈8.7	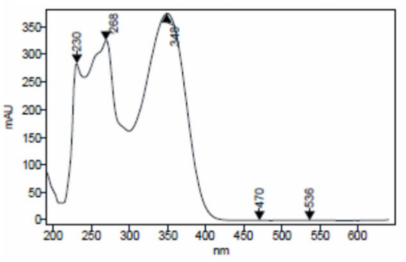

<table>
<tr><th>Compound</th><th>Retention time</th><th>UV spectra</th></tr>
<tr><td>Nothofagin</td><td>≈16.9</td><td>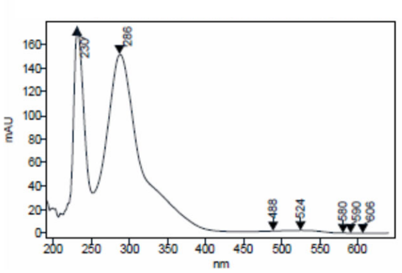
</td></tr>
<tr><td>Orientin</td><td>≈9.3</td><td>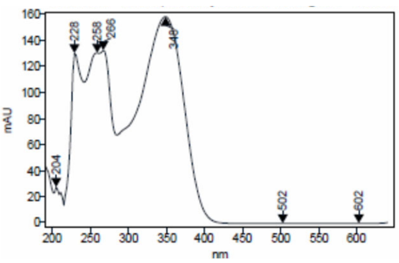
</td></tr>
<tr><td>Vitexin</td><td>≈12.8</td><td>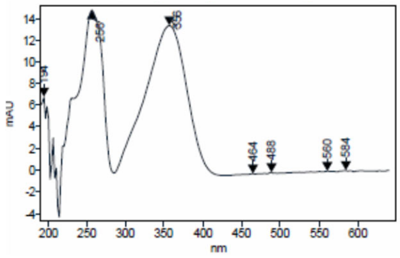
</td></tr>
<tr><td>Isovitexin</td><td>≈13.7</td><td>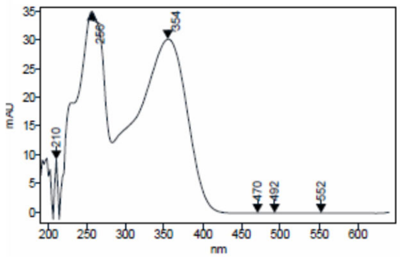
</td></tr>
<tr><td>Rutin</td><td>≈13.5</td><td>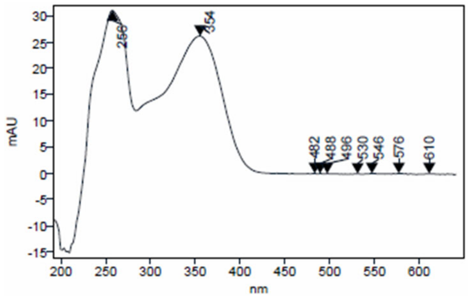
</td></tr>
<tr><td>Hiperoside</td><td>≈13.0</td><td>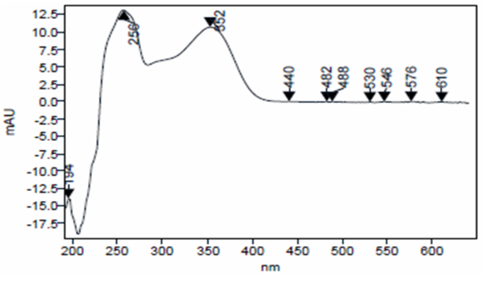
</td></tr>
</table>

Figure S7. Retention times and UV spectra of HPLC standards.

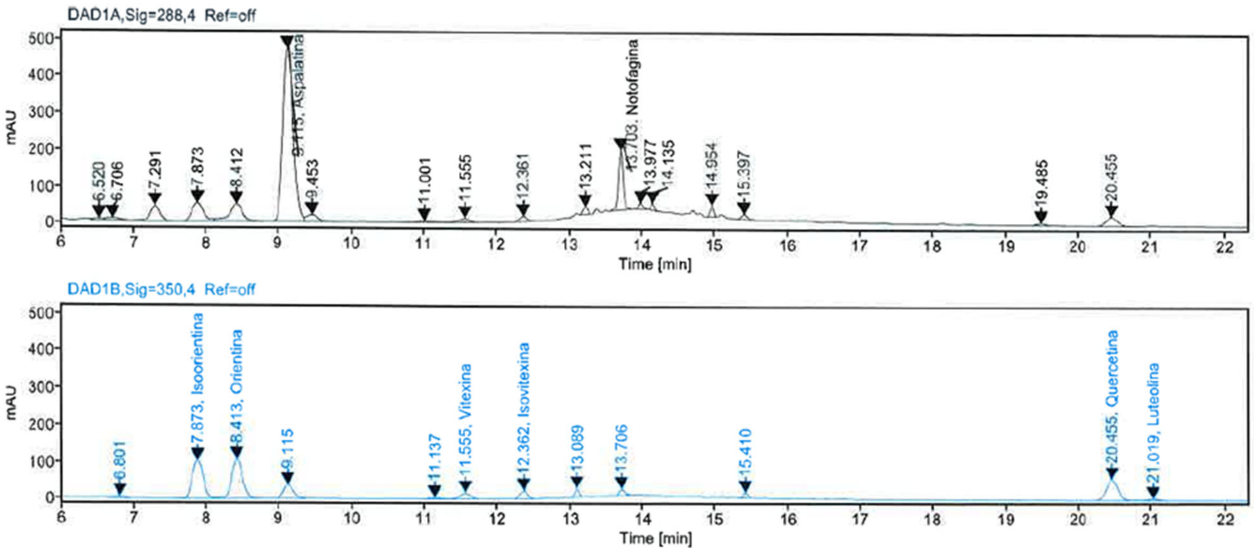

Figure S8. Chromatographic profile of ALU.

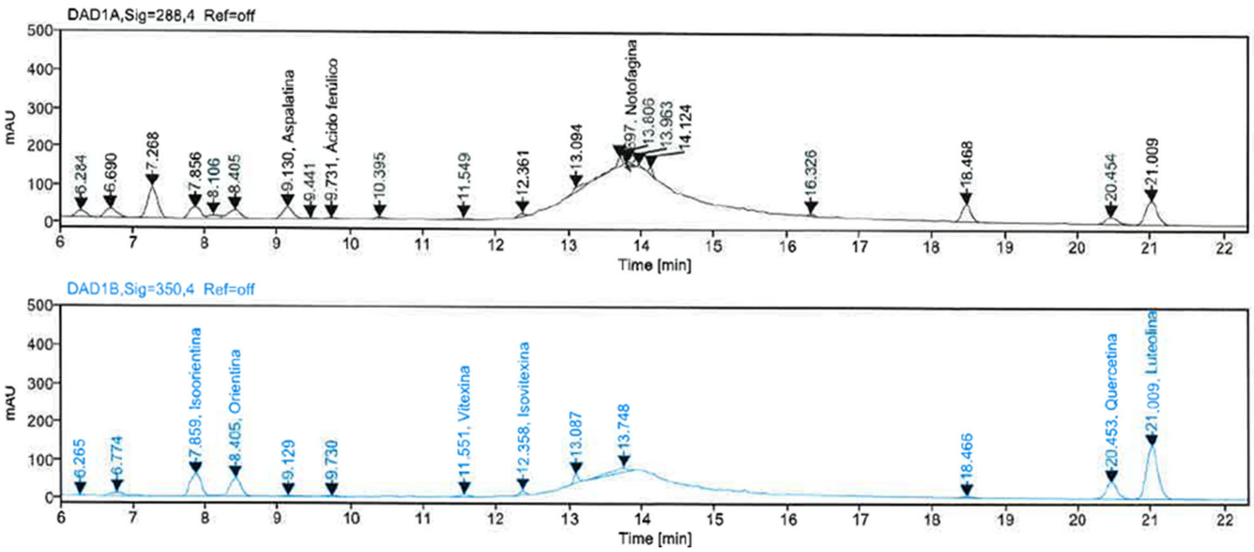

Figure S9. Chromatographic profile of ALF.
